# Methylglyoxal Induces Systemic Symptoms of Irritable Bowel Syndrome

**DOI:** 10.1371/journal.pone.0105307

**Published:** 2014-08-26

**Authors:** Shuang Zhang, Taiwei Jiao, Yushuai Chen, Nan Gao, Lili Zhang, Min Jiang

**Affiliations:** 1 Department of Gastroenterology, First Affiliated Hospital of China Medical University, Shenyang, China; 2 Department of Cadre Ward II, First Affiliated Hospital of China Medical University, Shenyang, China; University Hospital Llandough, United Kingdom

## Abstract

Patients with irritable bowel syndrome (IBS) show a wide range of symptoms including diarrhea, abdominal pain, changes in bowel habits, nausea, vomiting, headache, anxiety, depression and cognitive impairment. Methylglyoxal has been proved to be a potential toxic metabolite produced by intestinal bacteria. The present study was aimed at investigating the correlation between methylglyoxal and irritable bowel syndrome. Rats were treated with an enema infusion of methylglyoxal. Fecal water content, visceral sensitivity, behavioral tests and serum 5-hydroxytryptamine (5-HT) were assessed after methylglyoxal exposure. Our data showed that fecal water content was significantly higher than controls after methylglyoxal exposure except that of 30 mM group. Threshold volumes on balloon distension decreased in the treatment groups. All exposed rats showed obvious head scratching and grooming behavior and a decrease in sucrose preference. The serum 5-HT values were increased in 30, 60, 90 mM groups and decreased in 150 mM group. Our findings suggested that methylglyoxal could induce diarrhea, visceral hypersensitivity, headache as well as depression-like behaviors in rats, and might be the key role in triggering systemic symptoms of IBS.

## Introduction

Irritable bowel syndrome is one of the most frequently encountered disorders in outpatient gastroenterology practices, characterized by complex symptoms including abdominal pain and spasms, diarrhea, flatulence, altered bowel habits, headache, fatigue, loss of concentration, depression and heart palpitations, after excluding organic diseases [1]. Gastroenteric symptoms are associated with meals in almost two-thirds of patients suffering from IBS [2]. Elie Metchnikoff, who was awarded the 1908 Nobel Prize in Physiology or Medicine, proposed that bacteria in the colon could be the source of “toxicants” and toxic substances would lead to illness and aging [3]. A.K. Campbell and colleagues have made substantial progress in this theory. They indicated that carbohydrates not completely digested or absorbed in the small intestine reached the colon, where decomposed into hydrogen gas and other metabolites, methylglyoxal, methane, diacetyl, aldehydes and ketones [4].

Methylglyoxal has particular potential in all of the toxic metabolites [5]. Methylglyoxal is a natural substance in various organisms produced by the degradation of glycated proteins and monosaccharides [6], and detected in coffee, alcohol and foodstuffs. It is also a bacteria product from anaerobic glycolysis of carbohydrates in the large intestine. Previous studies have shown that methylglyoxal (0.1–10 mM) inhibits the growth of wild type *E. coli* cells via inducing rapid increase of cytosolic free Ca^2+^, followed by altered expression of at least 90 genes [7]. Moreover, several recent investigations have suggested that methylglyoxal is involved in many diseases such as diabetes, cancer, and obesity [8]–[10], and can also disrupt barrier function of brain microvascular endothelial cells [11]. These studies may provide a novel perspective on the pathogenesis of IBS. We therefore assumed that the toxic metabolites produced by gut bacteria might be the potential culprit for IBS. In the current study, we intended to investigate whether methylglyoxal had the potential to induce systemic symptoms in IBS via evaluations of abdominal reactions and behavioral tests as well as serum 5-HT level in rats, and to assess the evidence to clarify the association between methylglyoxal and IBS.

## Materials and Methods

### Ethics Statement

All procedures were approved by the Animal Care Committee of the Chinese Medical University and were in accordance with the principles outlined in the NIH Guide for the Care and Use of Laboratory Animals. All possible efforts were made to optimize the comfort and to minimize the use of the animals.

### Animals

Adult female Wistar rats weighing 180–200 g, were purchased from the Experimental Animal Center, China Medical University. Animals were housed in groups of 8 rats in polyethylene cages (L×W×H: 48×35×20 cm) on aspen chip bedding, containing wood chips and paper towels as enrichment. All animals were acclimatized under standard housing conditions (12/12 h light-dark cycle starting at 7:00 AM, temperature at 23±2°C, relative humidity at 50–60%) for 1 week before the experiment, with access to standard pelleted rodent chow (Trophic Animal Feed High-tech Co., Ltd., China) and tap water ad libitum. The treatments and experimental testing were conducted during the light component of the cycle. Any rough pelages and signs of diarrhea were noted.

### Treatments

The methylglyoxal stock solution (40%, Alfa Aesar, USA), which was freshly prepared before each experiment, was dissolved in saline and administrated as an enema (1 ml) at concentrations of 30 mM, 60 mM, 90 mM, 120 mM, and 150 mM. Rats were randomly divided into 6 groups (n = 8).The rats in the control group were treated with saline (1 ml).

### Fecal Water Content

Each rat was transferred into an individual metabolic cage on day 9 (1350×400×1500 mm), under where a separate metabolism tray was placed to observe the appearance of feces. Fecal samples were collected and thoroughly oven-dried (80°C, 48 h) to calculate fecal water content according to the equation: water content  =  100%×(wet weight − dry weight)/wet weight [12].

### Rectal Distension

Visceral sensitivity was assessed by measuring the abdominal withdrawal reflex (AWR) using a semiquantitative score. The rats were lightly anesthetized with ether after fasting for 12 h. Distension balloons were inserted through the anus of the rats and positioned 2 cm from the anal verge. The rats were then housed in transparent cages (200 mm×80 mm×80 mm) individually after woke up, in which they were not allowed to swivel but only able to move forward-backward. The balloon was distended with water at 37°C after one-hour adaptation, and the threshold intensity was observed by AWR test as previously reported with some modification [13].Visual observation of the animal response to ascending-limit distension was performed by blinded observers from minimum volume of 0.1 ml to maximum of 1.0 ml. The AWR score was assigned by blinded observers as follows: 0, no behavioral response to distension; 1, brief head movements followed by immobility; 2, contraction of abdominal muscle; 3, lifting of abdomen; 4, body arching and lifting of pelvic structure. The volumes on balloon distension were recorded when the rats reached an AWR score of 3 or more.

### Behavioral Testing

#### Head scratching and grooming

On day 10, the severity of headache was assessed by measuring head scratching and head grooming. The procedures were performed as described [14], [15]. The sum of movements were recorded, including head scratching, head grooming, washing the head and licking the fore paws. The behaviors were observed within 60 minutes after methylglyoxal administration.

#### Sucrose preference test (SPT)

SPT was conducted to evaluate depression-like behavior [16], [17]. All rats were first given weak (1%) sucrose solution in their home cage to reduce reaction to novel environment and to ensure stability of the experimental results. Training consisted of an initial 48 hours exposure to sucrose solution, followed by five 1-h tests in which sucrose was presented. On day 10, sucrose consumption was measured by weighing bottles before and after the test period. Sucrose preference was calculated as: 100%×(sucrose consumption)/(total fluid consumption).

### Analysis of Serum 5-Hydroxytryptamine (5-HT)

Blood samples were collected from decapitated rats between 9:00AM and 11:00AM twenty-four hours after behavioral testing and separated in a refrigerated centrifuge at 4°C (3,000 rpm×15 min). Serum was stored at −80°C until used. The serum 5-HT was quantified using enzyme linked immunosorbent assay (ELISA) kits (Quantikine; R&D Systems, Minneapolis, MN, USA) according to the manufacturer's instructions.

### Statistics

All data are presented as mean ± standard deviation. Parametric data were analyzed using the student's *t* test and ANOVA was used for multiple comparisons. Statistical analyses were performed using the SPSS 17.0 software. Results were considered to be statistically significant at *p*<0.05.

## Results

### Defecation

**Figure 1:**
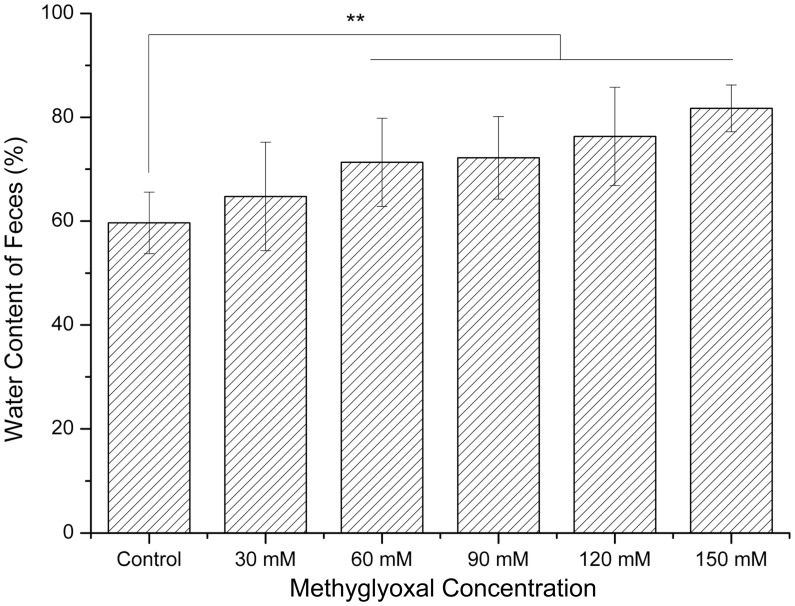
Effects of methylglyoxal on fecal water content. The fecal water content of 60 mM, 90 mM, 120 mM, 150 mM groups were significantly increased compared with controls. The 30 mM group showed no significant difference compared with controls. **p<0.01 versus controls.

Rat droppings of the control group were pellet shaped with a wet surface appearance and directly fell into metabolic tray without adhering to metabolic cage wall. However, feces adhered to the wall of metabolic cage in some rats of each treatment group. The time of rats first showing feces adhesion phenomenon in each group were day 4 (30 mM group), day 3 (60 mM group), day 3 (90 mM group), day 2 (120 mM group), day 2 (150 mM group), respectively. On day 10, the number of rats with feces adhesion in each treatment group was recorded, and the percentages of occurrence were 37.5% (30 mM group), 62.5% (60 mM group), 87.5% (90 mM group), 100% (120 mM group), 100% (150 mM group), respectively. None was observed in the control group. There were no significant differences in the fecal water content between the 30 mM group and controls (*p*>0.05) as shown in Figure 1. The fecal water contents of rats exposed to 60 mM, 90 mM, 120 mM and 150 mM methylglyoxal were significantly increased compared with controls (*p*<0.01) (Fig. 1).

### Visceral Sensitivity

**Figure 2:**
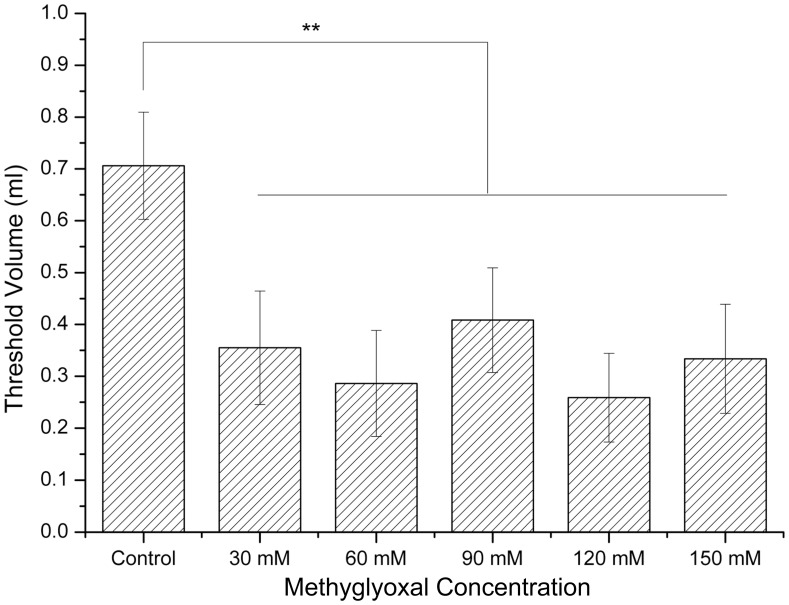
Effects of methylglyoxal on visceral sensitivity. The threshold volumes in all treatment groups were significant lower than controls. **p<0.01 versus controls.

The nociceptive threshold volume to elicit abdominal muscle contraction (AWR score is 3) was 0.70±0.10 ml in the control group. The mean threshold volumes in rats were 0.35 ml (30 mM), 0.28 ml (60 mM), 0.40 ml (90 mM), 0.25 ml (120 mM) and 0.33 ml (150 mM) (*p*<0.01) (Fig. 2).

### Head Scratching and Grooming

**Figure 3:**
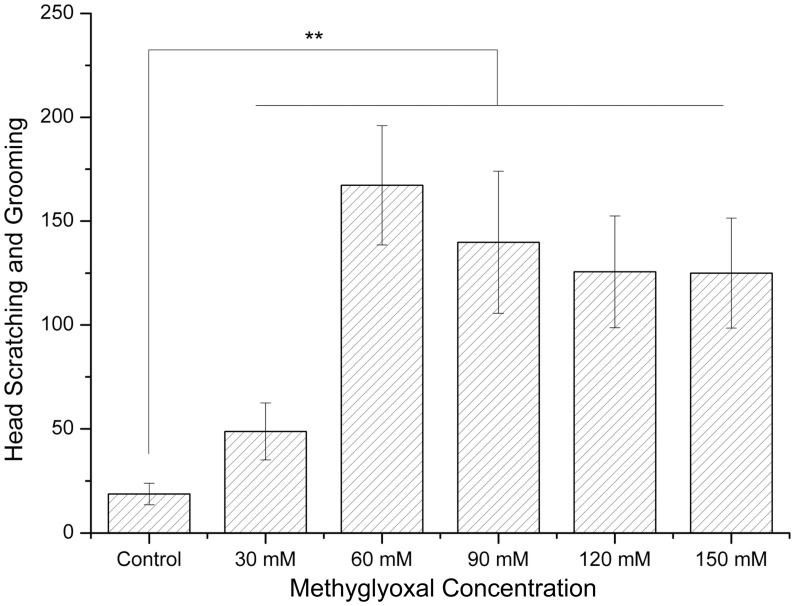
Effects of methylglyoxal on head scratching and grooming behaviors. The time of scratching behavior in all treatment groups markedly increased compared with controls. **p<0.01 versus controls.

All rats exposed to methylglyoxal performed more frequent head scratching and grooming activities than naive controls (*p*<0.01). The amount of activities were 167 (60 mM), 139 (90 mM) and 125 (120 mM). The behaviors showed significant negative correlation to the concentrations of methylglyoxal except 30 mM group (Fig. 3).

### Depression-Like Behaviors

**Figure 4:**
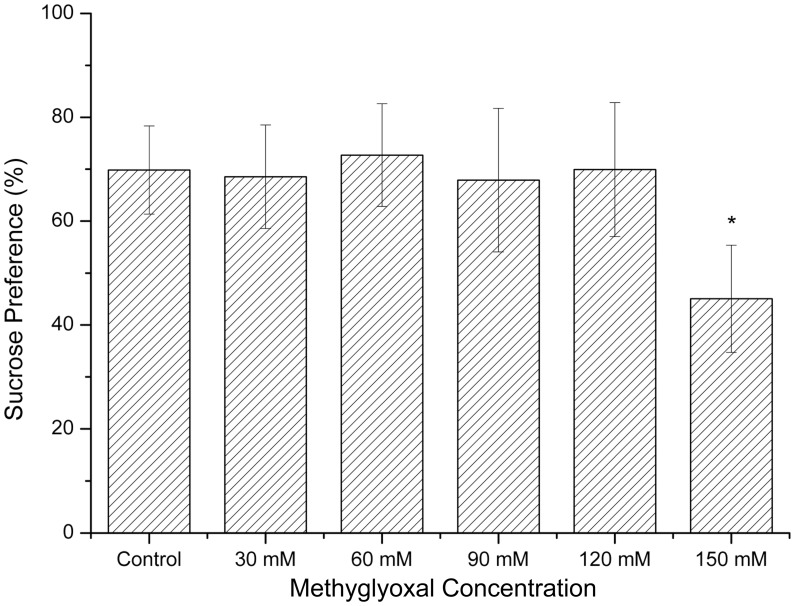
Effects of methylglyoxal on sucrose preference test. The SPT scores of the 150 mM group were decreased. *p<0.05 versus controls.

The sucrose preference of the rats treated with 150 mM methylglyoxal (45%) was significantly decreased compared with controls (69%) as shown in Figure 4 (*p*<0.01). However, the differences of the SPT between 30 mM, 60 mM, 90 mM, 120 mM groups and the control group were not significant (*p*>0.05).

### Serum 5-HT Levels

**Figure 5:**
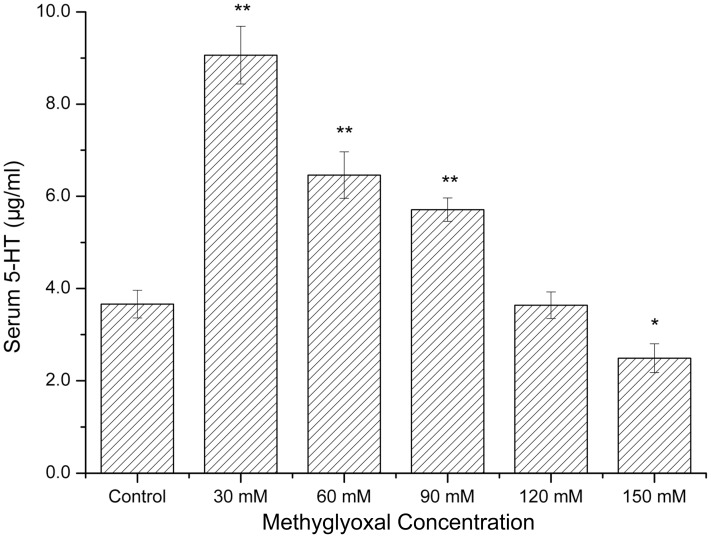
Effects of methylglyoxal on serum 5-HT level. The serum 5-HT levels of 30 mM, 60 mM and 90 mM groups were markedly higher than controls. The difference between 120 mM group and controls was not significantly. The level of 5-HT in 150 mM group rats was decreased than controls. **p<0.01 versus controls, *p<0.05 versus controls.

Rats exposed to 30 mM, 60 mM, 90 mM methylglyoxal presented significantly higher serum 5-HT levels than controls (*p*<0.01) with a dose-dependent decline. The serum level of 5-HT in 150 mM group was significantly decreased compared with the control group (*p*<0.01). There was no statistically significant difference between 120 mM group and controls (*p*>0.05) (Fig. 5).

## Discussion

IBS is the most common functional gastrointestinal disorder with worldwide incidence of up to 10%–20%. The clinical profile of patients with IBS is characterized by bowel symptoms such as abdominal pain, change in bowel habit, passing gas, accompanied by a range of other symptoms including tiredness, nausea, depression [18]. IBS is conventionally regarded as the outcome of a complex interaction between psychological and physical factors, however, a bacterial metabolic toxin hypothesis is now proposed, which postulates that gastrointestinal and systemic symptoms of IBS may be induced by intestinal bacterial metabolic toxins, the anaerobic products of carbohydrates not digested and absorbed in the small intestine [5]. The biological effects of methylglyoxal have been revealed in earlier studies. Cytotoxicity of methylglyoxal was investigated in some researches indicating severe inhibition of DNA, RNA and protein syntheses. The division of bacteria, cells in tissue culture and fertilized sea urchin eggs was inhibited by a low concentration of methylglyoxal [19]. It was reported to have pharmacological potential of antiviral, antimalarial and antibacterial activities [20]. Campbell pointed out that methylglyoxal could affect the intestinal microflora via calcium signaling, and also affect the signaling within the whole body. It offers a new perspective on the cause of intricate complex of symptoms of IBS.

The densities of microflora in the large intestine achieve concentrations of up to 10^11^or 10^12^ cells/g, and the composition pattern of an individual flora usually remains constant [21]. There are various functions of the normal gut microflora: fermentation of undigested food and endogenous mucus, supplement of short-chain fatty acids (SCFAs), participating in iron absorption, trophic effect on the intestinal epithelium, protection against pathogens, and influence on the homoeostasis of the immune system [22]. It is understood that the population of bacteria in lower gastrointestinal tract is much more complex than upper gastrointestinal tract. In patients with IBS, undigested and low-digestible carbohydrates which cannot be absorbed by the small intestine reach the large intestine, where contains little oxygen and large numbers of bacteria. Methylglyoxal and other intestinal bacteria metabolites, such as hydrogen and methane, are produced via anaerobic fermentation, suggesting why patients with IBS suffer from excessive gas and bloating. Our data showed that the fecal water content of rats treated with methylglyoxal was significantly higher than controls except the 30 mM group, and positively correlated with enemas concentration. During the observation on defecation, we noticed that the rat droppings were softer and more moisture in each treatment group than controls with the naked eye. Rat feces in the 120 mM and 150 mM groups were mushy and aqueous, and could not roll in the metabolic tray but stick to the bottom of the cage after discharging. Although there was no increase in fecal water content of the 30 mM group compared with controls, feces adhesion to the cage was also observed. Meanwhile this phenomenon had never been observed in the control rats, which suggested that methylglyoxal exposure had a positive effect on diarrhea in rats. It was shown that methylglyoxal (10 mM) induced contraction of guinea pig ileum in a standard organ bath preparation [5]. Stimulation of colonic transit determines insufficient time for intestine to absorb the water and leads to diarrhea. There is also a possibility that methylglyoxal induces diarrhea via alteration of intestinal microflora. Methylglyoxal is cytotoxic and growth of bacteria such as *E. coli* is inhibited by 0.2–1.2 mM methylglyoxal [23]. E. Mavric demonstrated that Manuka honey had notable antimicrobial activity which originates directly from the high level of methylglyoxal it contains [24]. The alteration of intestinal microbiota caused by methylglyoxal may depress the fermentation of SCFAs, and the decrease of SCFAs in the intestinal lumen will reduce water and sodium absorption in the colon and induce diarrhea eventually.

Serotonin (5-HT) is an essential monoamine neurotransmitter mainly located in the enterochromaffin (EC) cells lining the intestinal mucosa and the remainder is produced in central serotonergic neurons. Numerous studies have provided us with an in-depth understanding of the complex regulation of 5-HT on gastrointestinal functions. Abnormal regulation of 5-HT results in unusual motility and secretory activities of alimentary tract, diarrhea, visceral hypersensitivity, chronic constipation, and other gastrointestinal disorders. Previous studies have confirmed that methylglyoxal induces a rise in intracellular Ca^2+^ concentration by activating Ca^2+^channels in membrane of eukaryotic cells [25]–[27]. Ca^2+^ -dependent secretion of 5-HT in EC cells is mediated by activation of Ca^2+^ channels [28] and the release and secretion of 5-HT could be inhibited by calcium antagonist nifedipine [29]. Taken together, these findings suggest that methylglyoxal may stimulate 5-HT release by induction of Ca^2+^ influx in EC cells. Our results demonstrated that the serum levels of 5-HT were significantly increased in the 30, 60, 90 mM groups compared with controls (*p*<0.01), however, the difference between the 120 mM group and the control group was not significantly (*p*>0.05), while the serum level in the 150 mM group was lower than controls (*p*<0.01). It was consistent with the previous study, which showed that intracellular free calcium concentration was decreased with the increase of the concentration of methylglyoxal [30]. These data therefore reveal that methylglyoxal may modulate the serum 5-HT in a concentration-dependent manner. Serotonin is also known as a key agent that accelerates intestinal peristalsis in IBS via acting at specific serotonin-receptor subtypes. Nevertheless, the 5-HT level in rats treated with 30 mM methylglyoxal was significantly increased than all the other groups, and it implied that the 5-HT increase might not be the only trigger for diarrhea in rats. This situation could be interpreted as a combination of two factors, the toxic effects mediated by methylglyoxal including direct stimulation of intestinal motility and interfering with the ecological balance of the intestinal microflora, and the modulation of 5-HT. In the 30 mM group, stimulation of bowel movement due to increased 5-HT played a dominant role in the onset of diarrhea, while the toxic effects of methylglyoxal were secondary. In the 60 mM and 90 mM groups, the results suggested that the symptoms of the two groups were caused by both factors. The data of the 120 mM and 150 mM groups illustrated that diarrhea of the rats exposed to high concentrations of methylglyoxal was mainly maintained by the methylglyoxal toxicity.

Abdominal pain can be one of the most prominent symptoms of IBS and is attributed to visceral hypersensitivity. The data showed that the distension volumes to achieve an AWR score of 3 were remarkably lower in all rats exposed to methylglyoxal. A previous study revealed that visceral hypersensitivity involved the activation of spinal NMDA and non-NMDA receptors, meanwhile methylglyoxal could precisely induce the activation via a process which is discussed in the following passage. Another survey indicated that antibiotics could perturb intestinal microbiota, change the content of colon sensory neurotransmitter, and thus produce increased visceral sensitivity [31]. Investigation has shown that probiotics therapy prevents antibiotic-induced visceral hyperalgesia in mice [32]. Probiotics may locally control the nociceptive information transmitted to the intestinal nervous system by mediating the expression of receptors on epithelial cell such as opioid receptor and cannabinoid receptor type 2. Therefore, we infer that abdominal pain associated with IBS may result from methylglyoxal-induced visceral hypersensitivity and alterations of gut microflora.

A study of human neuroblastoma SH-SY5Y cells indicated that methylglyoxal was associated with the early plasma membrane depolarization and glutamate release and it could be prevented by N-methyl-d-aspartate (NMDA) receptors antagonists. The cell membrane was depolarized after a few minutes of exposure to methylglyoxal [33], followed by glutamate release, the relief of the Mg^2+^ block of NMDA receptors and Ca^2+^ influx, and afterwards it triggered a series of biochemical reactions in the neurons [34], and further changed the nature of the postsynaptic membrane and established the Long-term potentiation (LPT). Hippocampal LPT is involved in memory formation and consolidation. It may explain why patients with IBS exhibit symptoms of memory loss, poor concentration and other cognitive disorders. Glutamate is implicated in the development and maintenance of headache via interacting with its receptors [35]. The release of glutamate can activate the non-NMDA and NMDA receptors in spinal and supraspinal sites. The latter makes neurons more susceptible to nociceptive inputs and thus induces central sensitization, which is one of the putative mechanisms of headache [36], [37]. Our results were consistent with the notion in that head scratching and grooming were significantly induced in all treatment groups.

Depression is a mood disorder with a variety of causes. The main characteristics is pervasive and lasting low mood and it is a common symptom in patients with IBS [38]. The prevalence of depression was up to 37.1% with the OR of 6.3 in a recent survey [39]. A clinical research involved one hundred eleven individuals found that the Beck's inventory depression scores were significantly high in female patients with isolated fructose malabsorption and combined fructose/lactose malabsorption. The result was believed to be associated with increased fermentation of carbohydrates [40]. In support of this study, fructose elimination diet was found to improve symptoms of depression as well as gastrointestinal symptoms [41]. Likewise, probiotics could also alleviate the depression-like behaviors in adult mice [42]. The results in the present study showed that methylglyoxal might give rise to the occurrence of depression-like behaviors in rats. A newly proposed conceptual model of microbiota-gut-brain axis may explain the situation. Microbiota accesses the brain and influences behavior through bacterial products that gain access to the brain via the bloodstream, via the release of gut hormones such as 5-hydroxytryptamine (5-HT) from EC cells, via cytokine released from mucosal immune cells, or via afferent neural pathways [43]. Moreover, microbiota alters the expression of brain-derived neurotropic factor (BDNF), activity of Hypothalamic - pituitary-adrenal (HPA) axis, which has been confirmed to be closely related to depression-like behaviors [44], [45]. In support of the proposition, a probiotic, *Bifidobacteria infantis*, may be beneficial in the treatment of depression [46].

**Figure 6:**
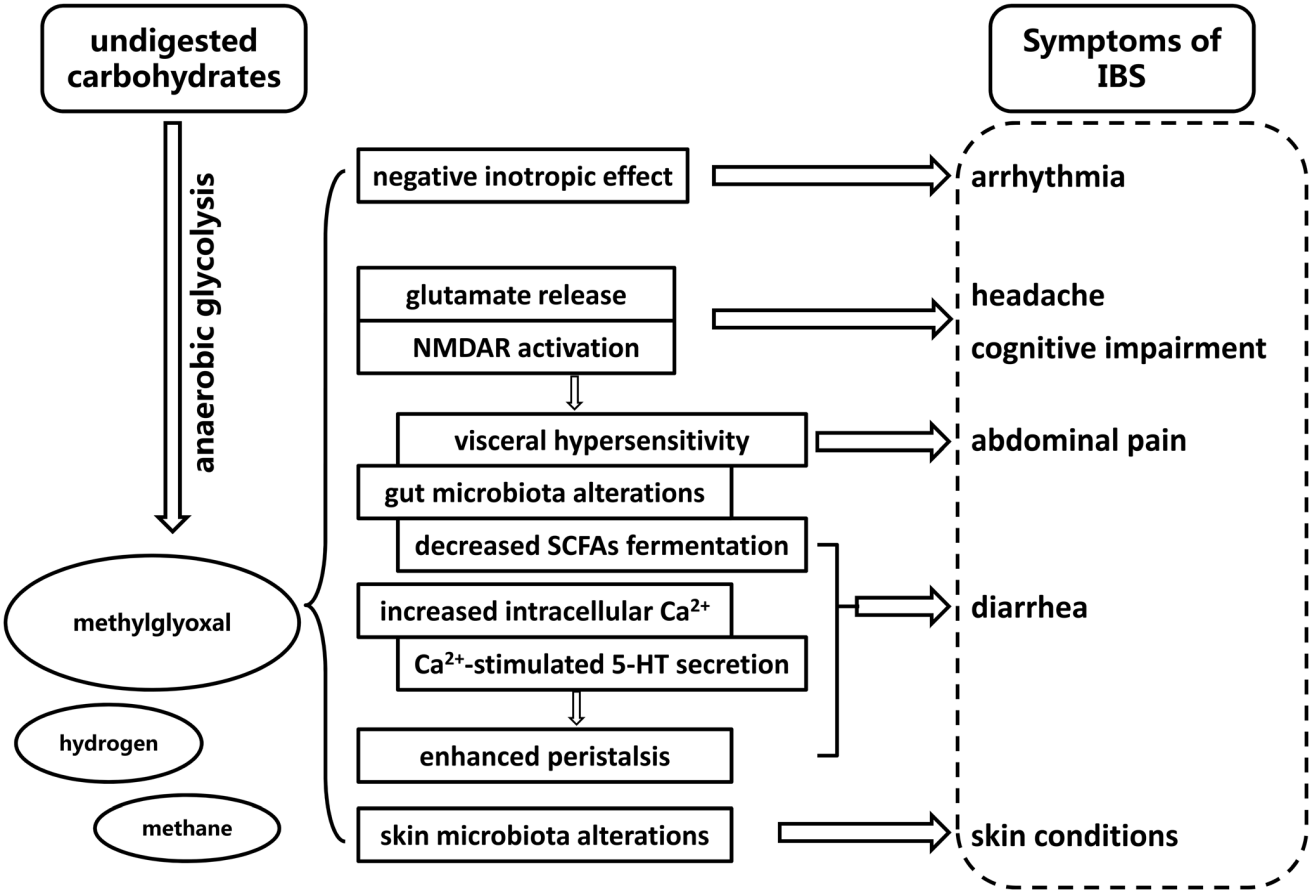
Diagram showing the mechanism that methylglyoxal induces various symptoms of IBS.

Studies have hitherto revealed potential link between methylglyoxal and other diseases. Methylglyoxal was observed to induce a major negative inotropic effect on the isolated perfused guinea pig heart in vitro, followed by a small positive inotropic effect [5]. Moreover, skin conditions in patients with IBS may be closely related to the skin microflora. Gueniche et al. indicated that alteration of the enteric microbiota through ingested probiotics showed beneficial effects on maintaining skin homeostasis after ultraviolet exposure [47]. The outline of our hypothesis is summarized and illustrated in Fig 6. The limitations of the current study are that we focus exclusively on diarrhea-predominant IBS and only one bacterial metabolite. Whether metabolites besides methylglyoxal can induce symptoms in constipation-predominant IBS and other subtypes requires further investigation.

In conclusion, the present finding suggests that a wide range of systemic symptoms of IBS including diarrhea, abdominal pain, headache, depression, cognitive impairment, arrhythmia and skin problems may be induced by methylglyoxal, an intestinal bacterial toxic metabolite. The result offers a hopeful target in the search for a unitary view on the etiology of IBS.

## Supporting Information

Checklist S1 ARRIVE Checklist.
(PDF)
